# The Effect of Arch Stiffness on the Foot–Ankle Temporal Kinematics during Gait Termination: A Statistical Nonparametric Mapping Study

**DOI:** 10.3390/bioengineering9110703

**Published:** 2022-11-17

**Authors:** Xuanzhen Cen, Peimin Yu, Yang Song, József Sárosi, Zhuqing Mao, István Bíró, Yaodong Gu

**Affiliations:** 1 Faculty of Sports Science, Ningbo University, Ningbo 315211, China; 2 Doctoral School on Safety and Security Sciences, Óbuda University, 1034 Budapest, Hungary; 3 Faculty of Engineering, University of Szeged, 6724 Szeged, Hungary; 4 Auckland Bioengineering Institute, The University of Auckland, Auckland 1010, New Zealand

**Keywords:** arch stiffness, gait termination, joint kinematics, metatarsophalangeal, SnPM

## Abstract

This study compares foot–ankle temporal kinematics characteristics during planned and unplanned gait termination (PGT and UGT) in subjects with different arch stiffnesses (ASs) based on the statistical nonparametric mapping (SnPM) method. By measuring three-dimensional arch morphological parameters under different loading conditions, 28 healthy male subjects were classified and participated in gait termination (GT) tests to collect metatarsophalangeal (MTP) and ankle-joint kinematics data. The two-way repeated-measures ANOVA using SnPM was employed to assess the impacts of AS on foot–ankle kinematics during PGT and UGT. Our results show that joint angles (MTP and ankle joints) were altered owing to AS and GT factors. The flexible arches hahadve periods of significantly greater MTP and ankle joint angles than those of stiff arches during the stance phase of GT, whereas subjects exhibited significantly smaller ankle and MTP joint angles during UGT. These results add additional insights into the morphological arch biomechanical function, and the comprehensive compensatory adjustment of lower-limb joints during gait stopping caused by unplanned stimulation.

## 1. Introduction

A well-functioning foot arch’s is significant in daily walking and running tasks [1,2]. As the primary structure for adjusting foot stiffness, the arch is springlike, as it compresses during the early stance phase and recoils during the late stance phase, which could improve gait efficiency by storing and returning mechanical work [1,3,4]. Given the stiffness of arch-spanning tissues, the windlass mechanism indicates that metatarsophalangeal (MTP) dorsiflexion produces the winding of the plantar fascia about the head of the metatarsus, thereby shortening and raising the arch, and inverting the subtalar joint [5]. On the other hand, considering the impact of altering MTP kinematics on the plantar fascia strain, the arch-spring mechanism further emphasizes the significant contribution of ligamentous structures, represented by the plantar fascia, to elastic energy absorption and dissipation [1]. Welte et al. [3] investigated the interaction between the above two mechanisms and found that the engagement of the windlass through MTP dorsiflexion reduced arch stiffness (AS), and increased energy storage and return. MTP dorsiflexion may consequently influence foot movement by adjusting the mechanical energy pattern. Kirsty et al. [6] also found that the plantar fascia demonstrated a characteristic elastic stretch-shortening cycle, with most of the strain produced through compressing the arch. The energy transfer mechanism of the plantar fascia between the MTP (energy absorption) and the foot arch (energy produced during recoil) reduces the strain required for the plantar fascia to produce positive mechanical work at the arch.

Structural changes in the foot arch unavoidably cause biomechanical alterations in the lower extremities, thus leading to impairments and musculoskeletal disorders of the foot and ankle [7,8,9,10]. Although arch height is overwhelmingly cited as a predictive factor for podiatry, there is emerging evidence that AS (or arch flexibility) might also be a critical contributor [11]. It is also considered to be a standard for evaluating injury susceptibility considering the association among ground reaction force (GRF), foot pronation/supination, and foot injury [11,12]. A comparative biomechanical study [4] analyzed the range of motion (ROM) of lower-limb joints (hip, knee, ankle, and MTP) during planned gait termination (PGT) and unplanned gait termination (UGT) in individuals with different ASs. The kinematic variations caused by differences in AS are mainly concentrated in the MTP and ankle joints. Despite these studies demonstrating links between arch morphological characteristics and discrete biomechanical data, little work has examined the correlation between AS and temporal kinematics. Statistical parametric mapping (SPM) has proven to be helpful in biomechanical data with time-varying characteristics in previous studies [13,14,15]. Statistical nonparametric mapping (SnPM), as an SPM nonparametric equivalent, permits hypothetical testing on the whole waveform rather than concentrating on specific data points, thus compensating for regional focus bias [16,17].

The amplitude of the loading on the arch would further increase in comparison to steady-state gait during gait termination (GT) [18]. Furthermore, the GT task was performed as a valuable tool for gait analysis, and it is widely used to assess motor function in patients with balance disorders [19,20,21]. As the closest anatomically to the arch, the biomechanical properties between the MTP and ankle joint are also worth exploring during GT induced by unplanned stimuli. Hence, this study uses an SnPM method to examine the foot–ankle temporal kinematic characteristics of stiff- and flexible-arched individuals during PGT and UGT. To remove a possible effect of sex and age, only young males were recruited to participate in the current study. Since more flexible arches tend to have a greater tendency to drop under load, we hypothesized that flexible arches would exhibit greater dorsiflexion angles in the sagittal plane during GT. Furthermore, subjects would have large ankle and MTP joint angles during UGT caused by unexpected stimulation in all motion planes.

## 2. Materials and Methods

### 2.1. Participants

Before the study, power analysis via G*Power version 3.1 software (Franz Faul, Kiel University, Kiel, Germany) for the current investigation was performed to calculate the minimal sample size (effect size = 0.25, alpha value = 0.05, and power value = 0.8). We recruited 28 male subjects and divided them into stiff-arch (SA, *n* = 14) and flexible-arch (FA, *n* = 14) groups on the basis of different ASs. The details of calculating the arch stiffness index (ASI) are presented in Section 2.2.1. All subjects had to meet the following recruitment standards: (1) healthy male with the right extremity as the dominant limb; (2) no lower extremity injuries or surgeries within the six months before data collection; (3) meeting the corresponding grouping criteria (SA: ASI > 1448 and FA: ASI < 1448) established by previous studies [4]. Before the experiment, informed consent was obtained from each subject, and the university ethics committee granted ethical approval.

### 2.2. Experimental Protocol and Procedure

#### 2.2.1. Foot Morphology Measurements

Compared with traditional foot dimensional measurements (e.g., digital caliper and digital footprint), three-dimensional (3D) foot morphological scanning for acquiring foot anthropometric parameters has relatively higher precision and robustness [22]. Therefore, before performing the gait termination task, 3D foot morphological parameters were collected from all participants during standing and sitting conditions using the Easy-Foot-Scan instrument (OrthoBaltic, Kaunas, Lithuania) following a previously developed protocol [18]. Participants’ foot morphology was only measured for their dominant feet. The variables of the foot structure were calculated with AutoCAD version 2018 software (Autodesk, San Rafael, CA, USA) according to the 3D foot images acquired from the foot morphology scanner in standing and sitting postures. The arch height index (AHI) in standing and sitting positions was calculated as the instep height divided by the foot length ball. The ASI of each participant was calculated by comparing the change in AHI between sitting and standing postures, and standardized to 40% body weight (BW), which reflects the change in the load placed on the feet under the two postures [11,23]. According to the categorization methodology, the SA (*n* = 14) and FA (*n* = 14) groups were recruited for the following gait termination task (Table 1).

#### 2.2.2. Gait Task Measurements

After completing foot morphological measurements, participants underwent a 5 min warm-up before gait task measurements. All participants were instructed to perform two types of GT tests, PGT and UGT, within a laboratory setting following a previously established protocol [4]. First, they were asked to walk barefoot along a 20 m walkway at a self-selected speed. If they received a transmitted auditory signal with a bell during this process, they needed to stop walking immediately and remain stationary. Otherwise, they had to stop systematically at a designated location at the end of the walkway. Of the GT tests, 20% included a ringing signal, while the remaining 80% did not. A 2 min rest interval was provided between trials to reduce the effect of tiredness on experimental results to a minimum. Each participant was requested to provide a dataset of 10 successful gait tests comprising 5 PGT tests and 5 UGT tests.

### 2.3. Data Acquisition and Processing

A motion capture system (Vicon Motion System Ltd., Oxford, UK) with eight infrared cameras was used to record kinematic data from the ankle and MTP joints of the dominant side at 200 Hz. On the basis of a previously established experimental design, 14 markers were placed on the surface of the participant to define the dominant shank, forefoot, and hindfoot [19]. The MTP joint in this study was defined as the angle between the forefoot and hindfoot anatomical coordinate systems [19].

An inverse kinematic algorithm was performed in Visual 3D version 3.26 software (C-Motion Inc., Germantown, MD, USA) to calculate MTP and ankle joint parameters in the sagittal, frontal, and transverse planes, and a second-order low-pass Butterworth filter with a cutoff frequency of 6 Hz was applied to denoise the marked trajectory. For each GT test, joint angles were adjusted on the basis of the stance phase and normalized to 101 time points.

### 2.4. Statistical Analysis

Due to the 1D time-varying properties of the ankle and MTP joint kinematics, a factorial SnPM was applied in MATLAB R2018a (The MathWorks, Natick, MA, USA) using open-access one-dimensional SPM scripts [16,24]. Two-way repeated-measures ANOVA was used to assess the effect of AS on foot–ankle kinematics during expected and unexpected gait terminations. Bonferroni correction was applied to adjust the alpha risk of post hoc tests in the case of significance. The level of statistical significance was set at *p* < 0.05.

## 3. Results

### 3.1. Ankle Kinematics

**Figure 1:**
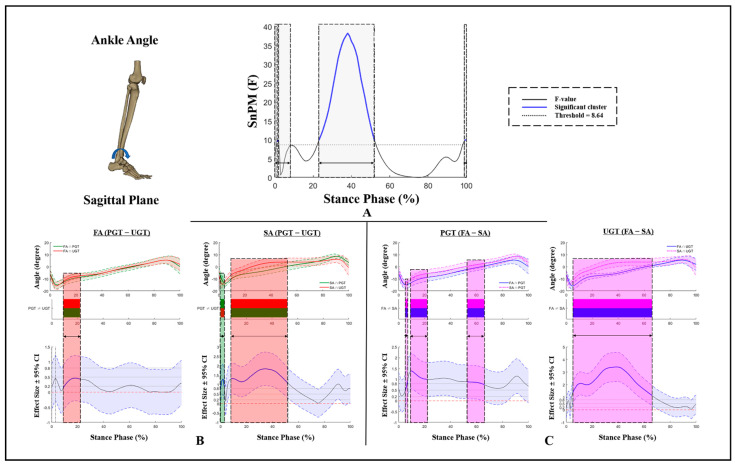
Kinematic differences of ankle angles in the sagittal plane. (**A**) Interaction, (**B**) post hoc test for GT, and (**C**) post hoc test for AS. SA: stiff arch; FA: flexible arch; PGT: planned gait termination; UGT: unplanned gait termination. Shaded gray vertical bars represent the areas where interaction effects exist. Shaded red and green vertical bars represent the area where data during UGT were significantly greater or smaller than during PGT. Shaded purple vertical bars represent the area where data for SA were significantly greater than for FA.

Kinematic differences in ankle angles in the sagittal plane are displayed in Figure 1. There was an interaction effect, with an F-value above the significant threshold of 8.64 during 23–51% of the stance phase (Figure 1A). Specifically, compared to PGT, FA and SA had significantly smaller ankle plantarflexion angles during 10–21% and 8–51% of the stance phase of UGT, respectively. Moreover, FA exhibited a significantly increased ankle plantarflexion angle in the sagittal plane during 10–21% and 53–65%, and 4–65% of the stance phase under PGT and UGT conditions, respectively (Figure 1B,C).

**Figure 2:**
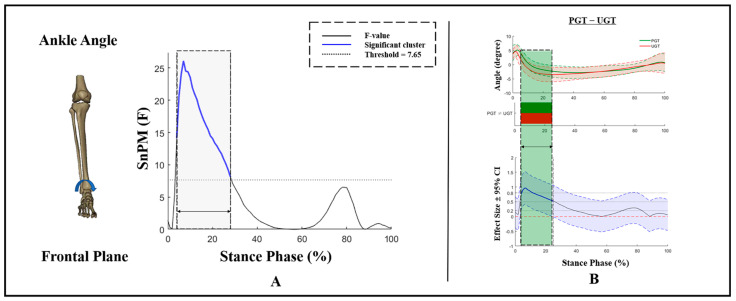
Kinematic differences of ankle angles in the frontal plane. (**A**) Main effects of GT and (**B**) post hoc test for GT. Shaded gray vertical bars represent the areas where main effects of GT exist. Shaded green vertical bars represent the area where data during UGT were significantly smaller than during PGT.

As for ankle angles in the frontal plane, no GT × AS interaction effects or main effects of AS were found (Figure 2). Additionally, at the early stance phase (4–25%), a GT effect with an F-value above the significant threshold of 7.65 presented (Figure 2A). During that phase, compared to PGT, a significantly greater ankle inversion angle was exhibited during UGT (Figure 2B).

**Figure 3:**
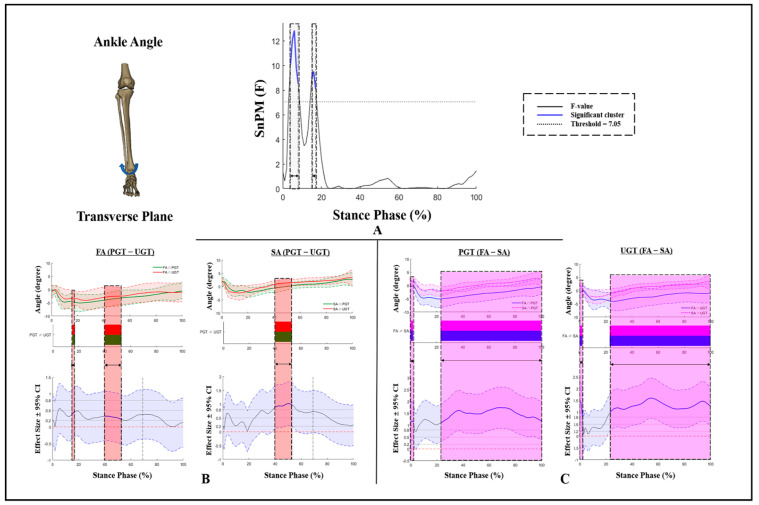
Kinematic differences of ankle angles in the transverse plane. (**A**) Interaction, (**B**) post hoc test for GT, and (**C**) post hoc test for AS. Shaded gray vertical bars represent the areas where interaction effects exist. Shaded red vertical bars represent the area where data during UGT were significantly greater than during PGT. Shaded purple vertical bars represent the area where data for SA were significantly greater than for FA.

Figure 3 exhibits differences in ankle angles in the transverse plane under different ASs and GTs. There were significant GT × AS interaction effects for ankle angles in the transverse plane during 4–6% and 16–18% of the stance phase. The ankle external rotation angles of FA and SA during 40–52% of PGT were significantly greater than those at UGT. Moreover, a significant decrease in the external rotation of SA during the stance phase of PGT and UGT (22–100%) was found (Figure 3B,C).

### 3.2. MTP Kinematics

As for MTP angles in the sagittal plane, no significant kinematic differences were found on the basis of the results of SnPM.

**Figure 4:**
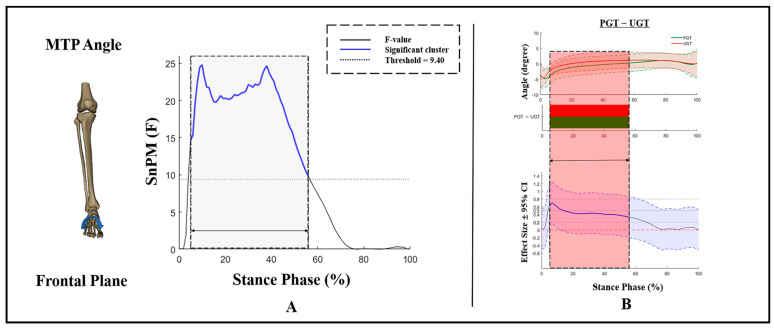
Kinematic differences of MTP angles in the frontal plane. (**A**) Main effects of GT and (**B**) post hoc test for GT. Shaded gray vertical bars represent the areas where the main effects of GT exist. Shaded red vertical bars represent the area where data during UGT were significantly greater than during PGT.

Kinematic differences in MTP angles in the frontal plane are exhibited in Figure 4, and no GT × AS interactions or main effects of AS were found during PGT and UGT. There was a GT effect with an F-value above the significant threshold of 9.4 during 4–57% of the stance phase (Figure 4A). During that phase, compared to PGT, a significantly smaller MTP inversion angle was exhibited during UGT (Figure 4B).

**Figure 5:**
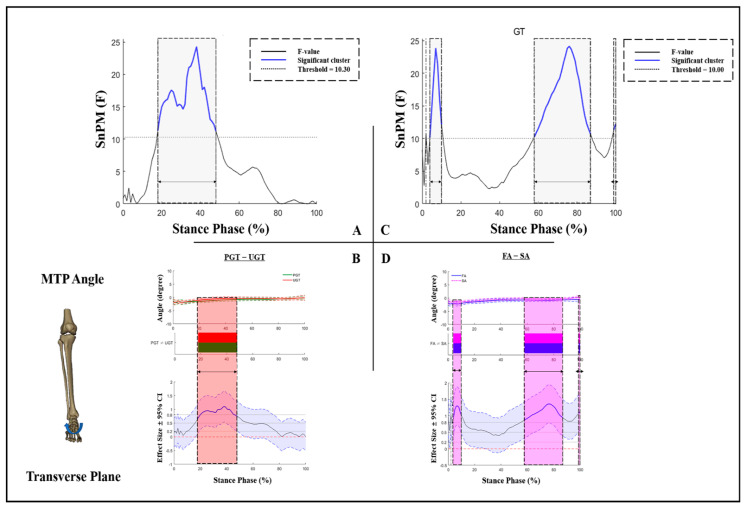
Kinematic differences of MTP angles in the transverse plane. (**A**) Main effects of GT and (**B**) post hoc test for GT; (**C**) main effects of AS and (**D**) post hoc test for AS. Shaded gray vertical bars represent the areas where the main effects of GT or AS exist. Shaded red vertical bars represent the area where data during UGT were significantly greater than during PGT. Shaded purple vertical bars represent the area where data for SA were significantly greater than for FA.

No GT × AS interaction effects were found for MTP angles in the transverse plane. Figure 5A shows a GT effect with an F-value above the significant threshold of 10.3 (18–48% of the stance phase). During that phase, compared to PGT, a significantly smaller MTP external rotation angle was exhibited during UGT (Figure 5B). During 4–10% and 58–87% of the stance phase, an AS effect with the F-value above the threshold of 10 was observed (Figure 5C). Moreover, FA exhibits a significantly increased MTP external rotation than SA (Figure 5D).

## 4. Discussion

The primary goal of this investigation was to compare the MTP and ankle kinematics during PGT and UGT across the entire waveform of joint angles between the FA and SA groups using an SnPM method. Overall, the hypotheses in the present study were partially correct, as the FA group has significantly greater MTP and ankle-joint angle periods than those of the SA group during the stance phase of GT. However, in contrast to our hypothesis, subjects exhibited significantly smaller ankle-joint angles in the three motion planes, and smaller MTP joint angles in the frontal and transverse planes during UGT.

The present study defined ASI as the change in AHI in the sitting and standing conditions, standardized to 40% BW [18]. Although not a dynamic index, the ASI is easily obtained and captures two phases of the foot load pattern (i.e., loading and unloading), thus indicating how the foot dynamically adapts to the load [11]. FAs tend to splay during the stance phase, and shift the load from the midfoot to the forefoot and rearfoot concurrently along the longitudinal axis of the foot. However, other studies noted that FAs exhibited a greater percentage of overall plantar impulse in the hindfoot than that of SAs [11,18]. The transfer of unilateral impulse on the foot longitudinal axis might be associated with the fact that, in the asymmetric and irregular triangular truss model formed by the plantar fascia and the arch bones, the shorter proximal side attached to the calcaneal tuberosities would suffer more impulses during arch compression [25,26]. Considering the relationship between AS and arch height (i.e., the low arch tends to be more flexible, while the high arch is more likely to be stiffer), FAs may stretch the soft tissues to create enough moment for toe-off during gait, while SAs may be less flexible and lack shock absorption [7,23].

Compared to steady-state gait, the arch receives an increase in the magnitude of load during GT, which can be inspected in the observable biomechanical parameters [18]. The experimental designs based on the GT model would allow for investigating the effect of morphological arch differences on foot–ankle biomechanics. As a multisegmental system, hip, knee, and ankle joint motion is associated with the lower limb kinetic chain; however, a previous study showed no significant effect of morphological differences in the foot arch on the kinematic compensation of proximal joints such as the hip and knee [4,27]. The FA group had significantly greater MTP and ankle-joint angle periods than those of the SA group during the stance phase of GT, which is also supported by the results found in this study. FA exhibits a significantly increased ankle plantarflexion in the sagittal plane during the braking and transitional phases of GT, while external rotation was significantly greater than that of SA during the transitional and stabilization phases. The elastic storage-return mechanism of the foot suggests that the human arch can compress when loaded, allowing for the storage of elastic strain energy [2,6]. The FA tends to splay along the longitudinal axis of the foot, resulting in the ankle joint exhibiting greater ROM (e.g., greater plantarflexion) in the sagittal plane during GT [4,11]. The more significant ankle external rotation of FA may be related to the morphology and function of the medial and lateral longitudinal arches, i.e., the former is higher, softer, and more flexible [25]. With the lateral longitudinal arch acting as weight-bearing support, the foot tends to compress the medial longitudinal arch, resulting in external rotation of the ankle joint. Similarly, the present experiment found a significant main effect of AS for MTP angles in the transverse plane, i.e., FA exhibited a greater external rotation of the MTP joint than that of SA. No significant differences in MTP angles in the sagittal plane were found according to the results of SnPM. The potential reasons for this may be related to the fact that the ROM of the MTP joint is limited in the sagittal plane due to its anatomical structure, although it may be smaller in the frontal and transverse planes [4]. However, the MTP movement in the sagittal plane, especially dorsiflexion, is the key driver [2]. The GT experiments designed in this study might have resulted in a significantly greater impact on the foot than that of a normal gait (e.g., walking and running) [18]. Therefore, during the stance phase of GT, both FA and SA present larger MTP dorsiflexion in a shorter termination time, approaching the maximal limit.

As a transitional motor task, GT involves the transition from cyclic gait to quiet standing, and experiments based on this transitional task can be designed to challenge both feedforward (i.e., PGT) and feedback neuromuscular control (i.e., UGT) [21,28]. Compared with PGT, subjects had significantly smaller ankle plantarflexion angles, greater inversion angles during the braking phase of UGT, and significantly smaller external rotation angles during the transitional phase (40–52%). The stimulus delay period is critical because subjects must determine whether they receive an unexpected stimulus to perform the appropriate gait termination strategy. The stimulus delay period is crucial since participants must determine whether they received an unexpected stimulus to execute the adequate GT strategy [29]. Once they capture the stopping indication during this phase, the body adopts a series of adjustments to create a net braking impulse via increasing the initial braking impulse and decreasing the push-off impulse during the braking phase [4,30,31]. The soleus amplitude activity could also be enhanced to moderate tibial progression, while the activity of the tibialis anterior and gluteus medius could be augmented to limit plantarflexion and maintain limb extension during the transitional phase [32]. Given the change in foot balance associated with GT patterns, these kinematic alterations could also lead to gait imbalance and an increased risk of joint injury [33,34,35]. As an essential contributor to lower-limb energetics, MTP requires energy storage, and creates little or no energy during braking and transitional phases [4,15,19]. Notably, significantly smaller MTP inversion and external rotation angles are exhibited during UGT during braking and transitional phases, which might be related to an integrated response concerning the MTP-ankle coordination pattern to compensate for increased ankle inversion [19,29,36].

There are some limitations of the present study that need to be acknowledged. First, this study revealed the foot–ankle temporal kinematics; while kinetic information was not presented, a future study should investigate kinetic changes in individuals with different arch morphological characteristics during GT. Moreover, while the SnPM was effective in ANOVA for biomechanical data with time-varying characteristics, post hoc tests with Bonferroni correction might be relatively approximate and conservative [16].

## 5. Conclusions

The present study provides insights into how individuals with SA or FA regulate foot–ankle kinematics during the stance phase of different GT patterns. Since a greater tendency to splay under loads, FA exhibited significantly larger MTP and ankle angles than those of SA during the stance phase of GT except in the MTP sagittal plane. During UGT induced by unknown stimuli, the lower extremity kinetic chain requires a comprehensive integration of the compensatory adjustment due to the increased urgency for the dynamic stability to be activated spontaneously. Our work gave a new understanding of the regulations of foot–ankle temporal kinematics during gait subtasks, and might be instructive to foot injury prediction and arch orthotics development.

## Figures and Tables

**Table 1:** Anthropometric characteristics of two groups (mean ± SD).

Characteristic	Total	Groups		*p*-Value
		SA	FA	
Number (n)	28	14	14	NA
Age (y)	23.53 ± 1.92	23.50 ± 1.99	23.79 ± 1.89	0.700
Height (m)	1.76 ± 0.03	1.76 ± 0.03	1.76 ± 0.04	0.592
Weight (kg)	68.88 ± 5.92	68.29 ± 5.22	69.07 ± 6.82	0.735
BMI (kg/m^2^)	22.17 ± 1.65	22.12 ± 1.64	22.18 ± 1.77	0.922
ASI	1466.42 ± 275.80	1244.93 ± 101.64	1734.86 ± 169.29	0.000 *

Note: BMI: body mass index; ASI: arch stiffness index; SA: stiff arches; FA: flexible arches; NA: not applicable. *, significant difference between the two groups, *p* < 0.05, independent-samples T-test in SPSS 25.0 (SPSS Inc., Chicago, IL, USA).

## Data Availability

The data presented in this study are available on request from the corresponding author. The data are not publicly available due to ethical considerations.
